# Supplementary material for: Zero- to One-Dimensional Zn24 Supraclusters: Synthesis, Structures and Detection Wavelength
Source: Nanomaterials (Basel). 2023 Nov 30;13(23):3058. doi: 10.3390/nano13233058 (PMC10707760; doi:10.3390/nano13233058)
Supplement: Supplementary file 1 [file nanomaterials-13-03058-s001.zip › nanomaterials-2727473-supplementary.pdf]

Supracluster Zn_{24} from 0D to 1D: synthesis, structures and Detection wavelength properties

Yating Chen [1,2], Zhonghang Chen [1], Jiming Wang [1], Xuandi Ma [1], Linyu Yuan [1], Shuhua Zhang [1,2,*] and Fushun Tang [1,*]

[1] Guangxi Key Laboratory of Electrochemical and Magnetochemical Functional Materials, College of Materials Science and Engineering, Guilin University of Technology, Guilin 541004, China; 1020180460@glut.edu.cn (Y.C.); 1020180495@glut.edu.cn (X.M.)
[2] College of Chemistry, Guangdong University of Petrochemical Technology, Maoming 525000, China
[*] Correspondence: zsh720108@163.com (S.Z.); tfushun@glut.edu.cn (F.T.)

Syntheses

1.1 Syntheses of L^nH_2 (n = 1-5)

1.1.1 Syntheses of L^1H_2

A mixture of 5-bromo-2-hydroxybenzaldehyde (20 mmol, 4.0180 g), 5-amino-1,2,3,4-tetrazole (Hatz, 20 mmol, 1.7050 g) and ethanol (20 mL) in a 100 mL flask refluxed at 353 K for 1 h. Beige precipitate appeared and then was rinsed three times with fresh ethanol (10 mL × 3) and dried at 50 °C for 24 h (yield: 5.254 g g, *ca*. 75 % based on Hatz). *Anal. Calc*. for L^1H_2: $C_8H_6N_5OBr$, (*M*r = 268.07), *calc.*: C, 35.83; H, 2.26; N, 26.13 %; Found: C, 35.75; H, 2.34; N, 26.22 %. IR data for L^1H_2 (KBr, cm^{-1}, Fig. S1†): 3378m, 1613s, 1556s, 1470 s, 1275 m, 1174 m, 1064 w, 728 w. ^1H NMR data (DMSO-d6, 400 MHz, Fig. S8†) are as follows: δ 10.06 (d, *J* = 11.1 Hz, 1H), 8.16 – 8.05 (m, 2H), 7.90 (dd, *J* = 6.6, 2.4 Hz, 1H), 6.46 (s, 2H).

1.1.2 Syntheses of L^2H_2

L^2H_2 was prepared in a similar way to L^1H_2, except that 5-bromo-2-hydroxy-benzaldehyde was replaced by 2-hydroxybenzaldehyde. Beige precipitate appeared and then was rinsed three times with fresh ethanol (10 mL × 3) and dried at 50 °C for 24 h (yield: 3.706 g, *ca*. 98 % based on Hatz). *Anal. Calc*. for L^2H_2: $C_8H_7N_5O$, (*M*r = 189.17), *calc.*: C, 50.77; H, 3.70; N, 37.02 %; Found: C, 50.74; H, 3.74; N, 37.05 %. IR data for L^2H_2 (KBr, cm^{-1}, Fig. S1†): 3416 w, 1613 s, 1476 m, 1270 m, 1057 s, 759 s. ^1H NMR data (DMSO-d6, 400 MHz, Fig. S9†) are as follows: δ 10.71 (s, 1H), 10.26 (d, *J* = 2.2 Hz, 1H), 7.69 – 7.49 (m, 1H), 7.07 – 6.90 (m,

2H), 6.47 (s, 3H).

1.1.3 Syntheses of L^3H_2

L^3H_2 was prepared in a similar way to L^1H_2, except that 5-bromo-2-hydroxy-benzaldehyde was replaced by 3-methoxy-2-hydroxybenzaldehyde. Beige precipitate appeared and then was rinsed three times with fresh ethanol(10 mL × 3) and dried at 50 °C for 24 h (yield: 4.165 g, *ca*. 95 %, based on Hatz). *Anal. Calc*. for L^3H_2: $C_9H_9N_5O_2$, (*M*r = 219.20), *calc.*: C, 49.27; H, 4.11; N, 31.93 %; Found: C, 49.24; H, 4.15; N, 31.96 %. IR data for L^3H_2 (KBr, cm^{-1}, Fig. S1†): 3421 w, 1603 s, 1531 m, 1461 w, 1257 m, 1066 w, 963 m, 762 m. ^1H NMR data (DMSO-d6, 400 MHz, Fig. S10†) are as follows: δ 10.25 (d, *J* = 11.4 Hz, 1H), 9.53 (s, 2H), 7.50 (d, *J* = 8.0 Hz, 2H), 7.23 (d, *J* = 7.8 Hz, 2H), 6.94 (dd, *J* = 16.8, 8.7 Hz, 2H).

1.1.4 Syntheses of L^4H_2

L^4H_2 was prepared in a similar way to L^1H_2, except that 5-bromo-2-hydroxy-benzaldehyde was replaced by 3-ethoxy-2-hydroxybenzaldehyde. Beige precipitate appeared and then was rinsed three times with fresh ethanol(10 mL × 3) and dried at 50 °C for 24 h (yield: 4.286 g, *ca*. 91.9 % based on Hatz). *Anal. Calc*. for L^4H_2: $C_{10}H_{11}N_5O_2$, (*M*r = 233.23), *calc.*: C, 53.75; H, 4.93; N, 31.35 %; Found: C, 53.68; H, 4.99; N, 31.42 %. IR data for L^4H_2 (KBr, cm^{-1}, Fig. S1†): 3435 w, 3412 s, 1600 s, 1464 s, 1251 s, 774 s. ^1H NMR data (DMSO-d6, 400 MHz, Fig. S11†) are as follows:δ 10.30 – 10.21 (m, 1H), 10.14 (s, 1H), 9.52 (s, 1H), 7.48 (d, *J* = 7.9 Hz, 1H), 7.22 (t, *J* = 7.4 Hz, 3H), 6.97 – 6.85 (m, 2H), 6.46 (s, 2H).

1.1.2 Syntheses of L^5H_2

L^5H_2 was prepared in a similar way to L^1H_2, except that 5-bromo-2-hydroxy-benzaldehyde was replaced by 5-chloro-2-hydroxybenzaldehyde. Beige precipitate appeared and then was rinsed three times with fresh ethanol (10 mL × 3) and dried at 50 °C for 24 h (yield: 4.339 g, *ca*. 97 % based on Hatz). *Anal. Calc*. for L^5H_2: $C_8H_6N_5OCl$ (*M*r = 223.62), *calc.*: C, 42.93; H, 2.68; N, 31.30 %; Found: C, 42.90; H, 2.72; N, 31.33 %. IR data for L^5H_2 (KBr, cm^{-1}, Fig. S1†): 3331 w, 1648 s, 1476 m,1269 s, 1059 s, 731 m. ^1H NMR data (DMSO-d6, 400 MHz, Fig. S12†) are as follows: δ 10.13 (s, 1H), 9.53 (s, 1H), 8.01 – 7.81 (m, 3H), 7.71 (d, *J* = 2.5 Hz, 1H).

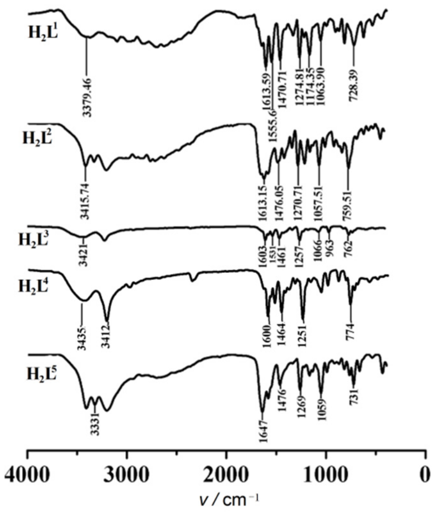

Figure S1. IR of H_2L^1-H_2L^5

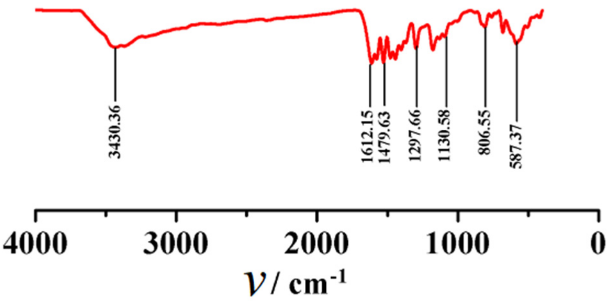

Figure S2. IR of **Zn_{24}**.

Table S1. Selected bond lengths (Å) and angles (°) for **Zn_{24}**

Zn9—N9	2.182(6)	Zn9—O30	2.004(6)
Zn9—N33	2.142(6)	Zn3—N10	2.117(6)
Zn9—N13	2.201(6)	Zn3—N34	2.206(6)
Zn9—O21	2.085(6)	Zn3—N11	2.211(6)
Zn9—N37	2.238(6)	Zn3—N17	2.209(7)
Zn3—O5	2.094(6)	Zn5—O8	2.002(7)
Zn3—O7	2.009(6)	Zn5—O9	2.079(6)
Zn5—N18	2.248(6)	Zn5—N44^{i}	2.141(6)
Zn5—N31	2.159(6)	Zn8—N32	2.163(6)
Zn5—N22	2.182(6)	Zn8—O16	2.063(6)
Zn8—O17	1.992(6)	Zn11—N8	2.173(6)
Zn8—N23	2.200(6)	Zn11—N4	2.210(7)
Zn8—N27	2.231(7)	Zn11—N38	2.224(6)
Zn8—N43^{i}	2.186(6)	Zn11—O29	2.003(8)
Zn11—N41	2.122(6)	Zn11—O28	2.037(7)
Zn2—N42	2.175(6)	Zn2—O18^{i}	2.019(6)
Zn2—N7	2.153(6)	Zn7—N26	2.043(7)
Zn2—N3	2.215(7)	Zn7—N24	2.053(7)
Zn2—O4	2.069(6)	Zn7—O15	1.929(8)
Zn2—N28^{i}	2.215(6)	Zn7—O13	2.143(8)

Zn7—O14	2.109(10)	Zn10—N14	2.034(7)
Zn6—N21	2.045(7)	Zn10—N36	2.029(7)
Zn6—N19	2.019(7)	Zn10—O22	1.914(8)
Zn6—O10	1.938(7)	Zn10—O23	1.998(10)
Zn6—O12	1.927(8)	Zn10—O24	2.436(17)
Zn6—O11	2.495(2)	Zn4—O5	2.818(7)
Zn4—N16	2.113(7)	Zn12—N5	2.057(8)
Zn4—N12	2.045(7)	Zn12—N39	2.080(8)
Zn4—O6	1.951(9)	Zn12—O27	1.946(9)
Zn4—O19	1.962(8)	Zn12—O26	2.223(8)
Zn4—O20	2.44(2)	Zn12—O25	2.061(14)
Zn1—N2	2.039(8)	Zn12—O28	2.818(9)
Zn1—N29^{i}	2.048(7)	Zn1—O3	1.937(9)
Zn1—O1	2.495(2)	Zn1—O2	2.021(11)
N33—Zn9—N13	89.9(2)		
N9—Zn9—N13	89.5(2)	N33—Zn9—N37	176.3(2)
N9—Zn9—N37	85.5(2)	O21—Zn9—N13	85.1(3)
N33—Zn9—N9	91.1(2)	O21—Zn9—N37	90.5(3)
N13—Zn9—N37	88.5(3)	O30—Zn9—N13	168.2(3)
O21—Zn9—N9	173.4 (3)	O30—Zn9—N37	93.1(3)
O21—Zn9—N33	92.7(3)	N10—Zn3—N34	90.0(2)
O30—Zn9—N9	102.3(3)	N10—Zn3—N11	90.5(2)
O30—Zn9—N33	89.2(3)	N10—Zn3—N17	175.8(2)
O30—Zn9—O21	83.2(3)	N17—Zn3—N11	88.5(2)
N34—Zn3—N11	89.1(2)	O5—Zn3—N10	93.3(3)
N34—Zn3—N17	85.9(2)	O5—Zn3—N34	171.4(3)
O5—Zn3—N34	171.4(3)	O7—Zn3—N10	89.5(3)
O5—Zn3—N11	82.9(3)	O7—Zn3—N34	103.0(3)
O5—Zn3—N17	90.7(3)	O8—Zn5—N18	92.7(3)
O7—Zn3—N11	167.9(3)	O8—Zn5—N31	99.4(3)
O7—Zn3—N17	92.3(3)	O9—Zn5—N18	92.4(2)
O7—Zn3—O5	85.0(3)	N32—Zn8—N23	90.6(2)
N31—Zn5—N18	86.8(2)	N32—Zn8—N27	177.6(2)
N31—Zn5—N22	90.2(2)	O17—Zn8—N32	88.3(2)
N22—Zn5—N18	89.4(2)	N23—Zn8—N27	88.7(2)
O8—Zn5—N22	170.2(3)	N43^{i}—Zn8—N23	88.8(2)
O8—Zn5—O9	84.3(3)	N41—Zn11—N4	90.8(2)
O8—Zn5—N44^{i}	88.9(2)	N32—Zn8—N43^{i}	90.2(2)
O9—Zn5—N44^{i}	89.9(3)	O16—Zn8—N32	92.6(3)
N44^{i}—Zn5—N18	177.4(2)	O16—Zn8—N23	84.4(3)
N44^{i}—Zn5—N31	90.8(2)	O16—Zn8—N27	89.7(3)
N44^{i}—Zn5—N22	89.3(2)	O16—Zn8—N43^{i}	172.6(2)
O29—Zn11—N38	92.2(3)	O17—Zn8—O16	86.0(3)
O29—Zn11—O28	84.5(4)	O17—Zn8—N23	170.3(2)
N43^{i}—Zn8—N27	87.4(2)	O17—Zn8—N27	92.8(3)
N41—Zn11—N8	90.9(2)	O17—Zn8—N43^{i}	100.9(2)
N41—Zn11—N38	176.5(3)	O28—Zn11—N41	91.9(3)
N8—Zn11—N4	89.5(2)	O28—Zn11—N8	173.1(3)
N8—Zn11—N38	85.8(2)	O28—Zn11—N4	84.2(4)

N4—Zn11—N38	88.0(2)	O28—Zn11—N38	91.2(3)
O29—Zn11—N41	89.6(3)	O4—Zn2—N3	88.2(3)
O29—Zn11—N8	101.8(3)	O4—Zn2—N28^{i}	91.5(3)
O29—Zn11—N4	168.7(3)	N28^{i}—Zn2—N3	89.1(3)
N42—Zn2—N3	89.2(2)	N24—Zn7—O14	133.1(5)
N7—Zn2—N42	91.0(2)	O15—Zn7—O14	111.7(6)
N7—Zn2—N3	89.0(2)	N19—Zn6—N21	99.5(3)
N7—Zn2—N28^{i}	177.3(3)	O10—Zn6—N21	108.8(3)
O4—Zn2—N42	177.0(3)	O12—Zn6—O10	109.8(4)
O4—Zn2—N7	90.4(3)	O22—Zn10—N14	114.6(4)
O18^{i}—Zn2—N42	96.9(3)	O22—Zn10—N36	104.9(4)
O18^{i}—Zn2—N7	87.6(3)	N16—Zn4—O20	139.1(6)
O18^{i}—Zn2—N3	173.0(3)	N12—Zn4—N16	96.6(3)
O18^{i}—Zn2—O4	85.7(3)	N12—Zn4—O20	93.6(5)
O18^{i}—Zn2—N28^{i}	94.4(3)	O6—Zn4—N16	98.8(4)
N26—Zn7—N24	99.9(3)	O6—Zn4—N12	121.3(4)
N26—Zn7—O13	135.6(3)	O19—Zn4—O20	50.5(6)
N26—Zn7—O14	90.5(4)	N5—Zn12—N39	95.6(3)
N24—Zn7—O13	91.5(3)	N5—Zn12—O26	97.9(3)
O15—Zn7—N26	112.8(4)	O27—Zn12—O26	97.2(3)
O15—Zn7—N24	106.0(4)	O27—Zn12—O25	98.7(6)
O15—Zn7—O13	104.7(3)	O25—Zn12—N39	92.1(4)
O14—Zn7—O13	53.0(3)	O2—Zn1—N29^{i}	95.7(4)
O10—Zn6—N19	111.6(3)	N2—Zn1—N29^{i}	100.2(3)
O12—Zn6—N21	98.7(3)	O3—Zn1—N2	105.3(4)
O12—Zn6—N19	125.8(4)	O3—Zn1—O2	104.7(5)
N14—Zn10—O24	143.5(4)	O3—Zn1—N29^{i}	111.6(4)
N36—Zn10—N14	99.3(3)	O2—Zn1—N2	137.7(5)
N36—Zn10—O24	91.3(4)	N5—Zn12—O25	135.9(6)
O22—Zn10—O23	111.2(6)	N39—Zn12—O26	144.6(3)
O22—Zn10—O24	95.8(5)	O27—Zn12—N5	121.4(4)
O23—Zn10—N14	97.1(4)	O27—Zn12—N39	103.3(4)
O23—Zn10—N36	129.0(6)	O25—Zn12—O26	56.1(5)
O23—Zn10—O24	51.2(5)	O19—Zn4—N16	93.9(4)
O6—Zn4—O19	106.1(4)	O19—Zn4—N12	128.9(4)
O6—Zn4—O20	109.2(6)		

Symmetry codes: (i) −*x*, −*y*, −*z*.

Table S2. Selected bond lengths (Å) and angles (°) for **1-D⊂Zn$_{24}$**

Zn1—O2	1.83(2)	Zn1—O3	1.908(17)
Zn1—O3′	1.962(17)	Zn1—N1	2.055(7)
Zn1—O2′	2.092(17)	Zn1—N11	2.120(7)
Zn1—O33	2.341(10)	Zn2—O5	2.039(7)
Zn2—N6	2.099(6)	Zn2—N17	2.195(6)
Zn2—N12	2.197(6)	Zn2—N2	2.211(6)
Zn3—O6	1.998(6)	Zn3—O9	2.093(6)
Zn3—N26	2.125(6)	Zn3—N18	2.161(6)
Zn3—N22	2.178(6)	Zn3—N13	2.264(6)
Zn4—O10	1.927(7)	Zn4—O7′	1.986(18)

Zn4—N14	2.002(6)	Zn4—O8	2.01(3)
Zn4—N21	2.073(7)	Zn5—O15	1.956(6)
Zn5—O14	2.030(7)	Zn5—N24	2.088(7)
Zn5—O30^{ii}	2.129(6)	Zn5—N31	2.214(6)
Zn6—O11	2.027(5)	Zn6—O13	2.102(6)
Zn6—N19	2.130(6)	Zn6—N32	2.171(6)
Zn6—N27	2.224(6)	Zn6—N23	2.233(6)
Zn7—O12	2.008(6)	Zn7—N17	2.092(6)
Zn7—N9^{i}	2.149(6)	Zn7—N28	2.162(6)
Zn7—N38	2.179(6)	Zn7—N33	2.211(6)
Zn8—O19	1.92(2)	Zn8—O18	1.936(6)
Zn8—O20^{i}	1.94(2)	Zn8—N39	2.030(7)
Zn8—N34	2.054(6)	Zn8—O20	2.48(2)
Zn9—O22^{i}	1.932(15)	Zn9—O23	1.939(6)
Zn9—O21	2.01(3)	Zn9—N44	2.050(6)
Zn9—N36	2.050(6)	Zn10—O25	2.015(6)
Zn10—O24	2.077(6)	Zn10—N29	2.131(6)
Zn10—N8^{i}	2.165(6)	Zn10—N43	2.201(6)
Zn10—N37	2.232(6)	Zn11—O26	2.021(6)
Zn11—O27	2.033(6)	Zn11—N3^{i}	2.162(6)
Zn11—N16^{i}	2.179(6)	Zn11—N7^{i}	2.214(6)
Zn11—N42	2.220(6)	Zn12—O28	2.000(7)
Zn12—O29	2.066(6)	Zn12—N41	2.129(6)
Zn12—N4^{i}	2.151(7)	Zn12—O32	2.209(6)
Zn12—O31	2.222(7)		
O2—Zn1—O3	108.6 (9)	O2—Zn1—N1	115.7 (7)
O3—Zn1—N1	128.1 (7)	O3′—Zn1—N1	96.2 (6)
O3′—Zn1—O2′	124.5 (7)	N1—Zn1—O2′	124.5 (7)
N1—Zn1—O2′	130.3 (5)	O2—Zn1—N11	112.4 (7)
O3—Zn1—N11	92.1 (5)	O3′—Zn1—N11	114.7 (6)
N1—Zn1—N11	94.9 (3)	O2′—Zn1—N11	92.4 (5)
O2—Zn1—O33	58.6 (10)	O3—Zn1—O33	98.2 (9)
O3′—Zn1—O33	77.2 (10)	N1—Zn1—O33	83.4 (8)
O2′—Zn1—O33	168.1 (8)	O6—Zn3—O9	81.9 (3)
O6—Zn3—N26	88.7 (3)	O9—Zn3—N26	90.1 (2)
O6—Zn3—N18	99.8 (3)	O9—Zn3—N18	178.1 (2)
N26—Zn3—N18	90.9 (2)	O6—Zn3—N22	169.3 (3)
O9—Zn3—N22	87.6 (3)	N26—Zn3—N22	89.6 (2)
N18—Zn3—N22	90.8 (2)	O6—Zn3—N13	93.9 (2)
O9—Zn3—N13	93.6 (2)	N26—Zn3—N13	175.8 (2)
N18—Zn3—N13	85.4 (2)	N22—Zn3—N13	88.5 (2)
O10—Zn4—O7′	95.4 (6)	O10—Zn4—N14	114.3 (3)
O10—Zn4—O8	127.2 (7)	O7′—Zn4—N14	115.7 (7)
O7′—Zn4—O8	104.0 (3)	O7′—Zn4—N21	128.4 (7)
N14—Zn4—N21	99.0 (3)	O8—Zn4—N21	107.4 (7)
O15—Zn5—O14	108.2 (3)	O15—Zn5—N24	120.2 (3)
O14—Zn5—N24	131.6 (2)	O15—Zn5—O30^{ii}	102.1 (3)
O14—Zn5—O30^{ii}	87.7 (3)	N24—Zn5—O30^{ii}	84.0 (2)
O15—Zn5—N31	87.4 (3)	N24—Zn5—N31	92.2 (2)

O30^{ii}—Zn5—N31	169.3 (2)	O11—Zn6—O13	86.9 (2)
O11—Zn6—N19	90.1 (2)	O13—Zn6—N19	94.0 (2)
O11—Zn6—N32	91.8 (2)	O13—Zn6—N32	89.6 (2)
N19—Zn6—N32	176.0 (2)	O11—Zn6—N27	104.3 (2)
O13—Zn6—N27	167.9 (2)	N19—Zn6—N27	90.5 (2)
N32—Zn6—N27	85.6 (2)	O11—Zn6—N23	167.9 (2)
O13—Zn6—N23	81.0 (2)	N19—Zn6—N23	90.3 (2)
N32—Zn6—N23	88.6 (2)	N27—Zn6—N23	87.8 (2)
O12—Zn7—O17	85.6 (3)	O12—Zn7—N9^{i}	88.7 (2)
O17—Zn7—N9^{i}	90.7 (2)	O12—Zn7—N28	95.1 (3)
O17—Zn7—N28	178.0 (2)	N9^{i}—Zn7—N28	91.1 (2)
O12—Zn7—N38	172.6 (2)	O17—Zn7—N38	87.8 (2)
N9^{i}—Zn7—N38	88.0 (2)	N28—Zn7—N38	91.5 (2)
O12—Zn7—N33	95.2 (2)	O17—Zn7—N33	90.9 (2)
N9^{i}—Zn7—N33	175.8 (2)	N28—Zn7—N33	87.2 (2)
N38—Zn7—N33	88.2 (2)	O19—Zn8—O18	117.8 (6)
O18—Zn8—O20′	111.0 (7)	O19—Zn8—N39	121.1 (7)
O18—Zn8—N39	113.9 (2)	O20′—Zn8—N39	97.9 (6)
O19—Zn8—N34	93.0 (5)	O18—Zn8—N34	106.2 (3)
O20′—Zn8—N34	128.7 (7)	N39—Zn8—N34	98.1 (3)
O19—Zn8—O20	58.9 (7)	O18—Zn8—O20	92.2 (5)
N39—Zn8—O20	151.6 (5)	O22′—Zn9—O23	115.3 (6)
O23—Zn9—O21	103.8 (7)	O22′—Zn9—N44	97.7 (5)
O23—Zn9—N44	117.0 (3)	O21—Zn9—N44	133.0 (8)
O22′—Zn9—N36	125.4 (7)	O23—Zn9—N36	102.9 (3)
O21—Zn9—N36	94.7 (8)	N44—Zn9—N36	97.9 (3)
O25—Zn10—O24	82.5 (2)	O25—Zn10—N29	89.8 (2)
O24—Zn10—N29	89.2 (2)	O25—Zn10—N8^{i}	104.6 (2)
O24—Zn10—N8^{i}	172.9 (2)	N29—Zn10—N8^{i}	91.6 (2)
O25—Zn10—N43	90.8 (2)	O24—Zn10—N43	92.1 (2)
N29—Zn10—N43	178.6 (2)	N8^{i}—Zn10—N43	87.1 (2)
O25—Zn10—N37	167.8 (2)	O24—Zn10—N37	85.3 (2)
N29—Zn10—N37	90.2 (2)	N8^{i}—Zn10—N37	87.7 (2)
N43—Zn10—N37	89.5 (2)	O26—Zn11—O27	84.4 (3)
O26—Zn11—N3^{i}	172.8 (2)	O27—Zn11—N3^{i}	91.5 (3)
O26—Zn11—N16^{i}	85.1 (2)	O27—Zn11—N16^{i}	89.1 (2)
N3^{i}—Zn11—N16^{i}	88.9 (2)	O26—Zn11—N7^{i}	93.1 (3)
O27—Zn11—N7^{i}	177.1 (2)	N3^{i}—Zn11—N7^{i}	90.8 (2)
N16^{i}—Zn11—N7^{i}	89.2 (2)	O26—Zn11—N42	95.6 (2)
O27—Zn11—N42	95.1 (2)	N3^{i}—Zn11—N42	90.7 (2)
N16^{i}—Zn11—N42	175.8 (2)	N7^{i}—Zn11—N42	86.6 (2)
O28—Zn12—O29	167.7 (3)	O28—Zn12—N41	97.7 (3)
O29—Zn12—N41	87.8 (3)	O28—Zn12—N4^{i}	100.6 (3)
O29—Zn12—N4^{i}	89.7 (3)	N41—Zn12—N4^{i}	95.5 (3)
O28—Zn12—O32	88.2 (3)	O29—Zn12—O32	85.2 (2)
N41—Zn12—O32	171.3 (2)	N4^{i}—Zn12—O32	89.6 (3)
O28—Zn12—O31	83.5 (3)	O29—Zn12—O31	85.6 (3)
N41—Zn12—O31	88.9 (3)	N4^{i}—Zn12—O31	173.4 (3)

O32—Zn12—O31	85.4 (2)

Symmetry codes: (i) −x+1, −y+1, −z+1; (ii) x, y, z+1; (iii) x, y, z−1.

Table S3 Hydrogen bond lengths (Å) and angles (°) for **Zn_{24}**

D—H···*A*	*D*—H	H···*A*	*D*···*A*	*D*—H···*A*
N1—H1A···O1	0.8600	2.1300	2.9292	155.00
N1—H1B···O26	0.8600	2.3900	3.1249	144.00
N6—H6A···O7	0.8600	2.3100	2.8830	124.00
N6—H6B···O4	0.8600	2.4300	3.0666	131.00
N6—H6B···O18	0.8600	2.4600	2.9979	121.00
N15—H15A···O20	0.8600	2.0700	2.8696	154.00
N15—H15B···O23	0.8600	2.2100	2.9593	146.00
N20—H20A···O19	0.8600	2.1300	2.8842	146.00
N20—H20B···O11	0.8600	2.0400	2.8427	156.00
N25—H25A···O13	0.8600	2.0500	2.8121	148.00
N25—H25B···$O12^{i}$	0.8600	2.2500	3.0047	146.00
N30—H30A···O14	0.8600	1.9400	2.7042	147.00
N30—H30B···O2	0.8600	2.1300	2.8987	148.00
N35—H35A···$O17^{i}$	0.8600	2.3600	2.9156	123.00
N35—H35B···O21	0.8600	2.5800	3.2182	132.00
N35—H35B···O30	0.8600	2.3800	2.9499	125.00
N40—H40A···O25	0.8600	2.0900	2.8396	145.00
N40—H40B···O24	0.8600	2.0300	2.8168	152.00
N45—H45A···O29	0.8600	2.2500	2.8589	128.00
N45—H45B···O9	0.8600	2.5000	3.0955	128.00
N45—H45B···$O8^{i}$	0.8600	2.3500	2.9337	125.00

Symmetry code: (i) $-x+1, -y+1, -z+1$.

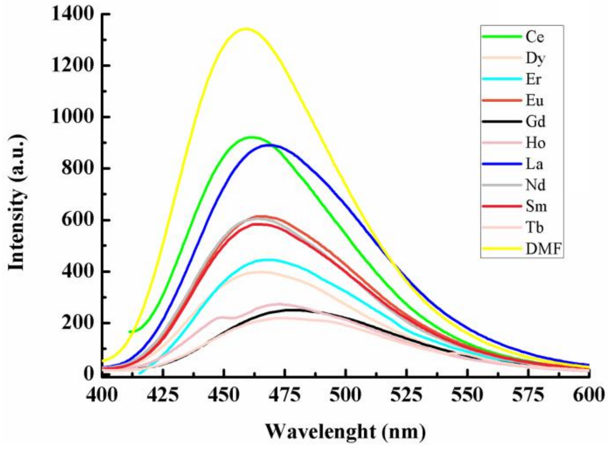

Figure S3. The liquid-state fluorescence behaviors of **Zn_{24}** in DMF.

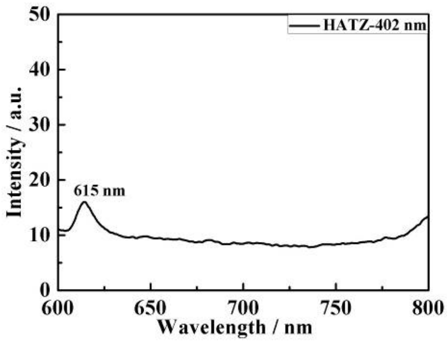

Figure S4. Emission spectra of HATZ in a solid state at 402 nm excitation wavelength at room temperature.

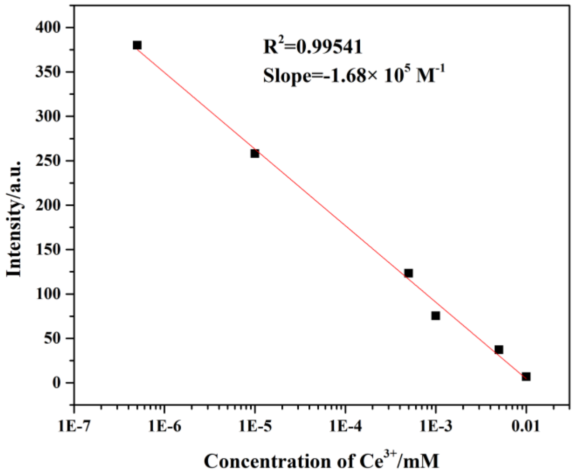

Linear Equation: y = -167.880 x – 86.209; R^2 = 0.9954; Slope = -1.68× 10^5 $M^{-1;}$ δ = 4.77 (N=12)

Figure S5 The fitting curve of the luminescence intensity of **Zn24** at different Ce^{3+} concentration

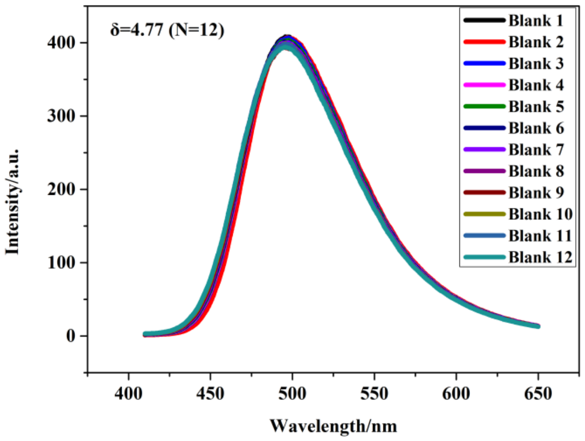

Figure S6 The fluorescence spectra of blank **Zn24** (1 mg·mL^{-1}) at different measurements.

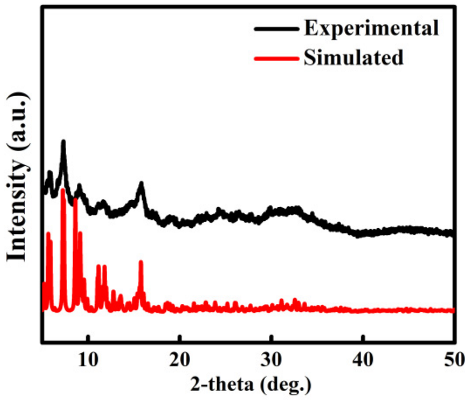

Figure S7 XRD of the complex **Zn_{24}**

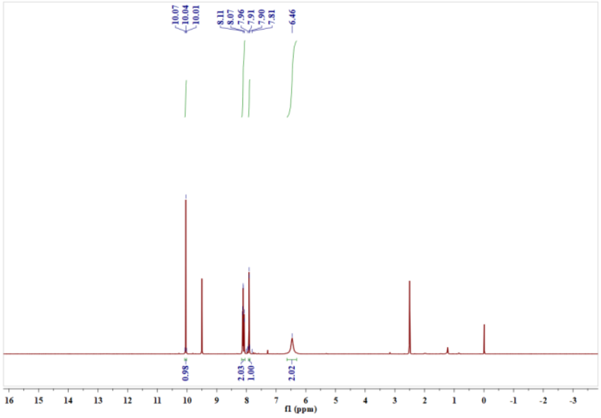

Figure S8 ^{1}H NMR (400MHz, DMSO-d_6) for **L^1H_2**.

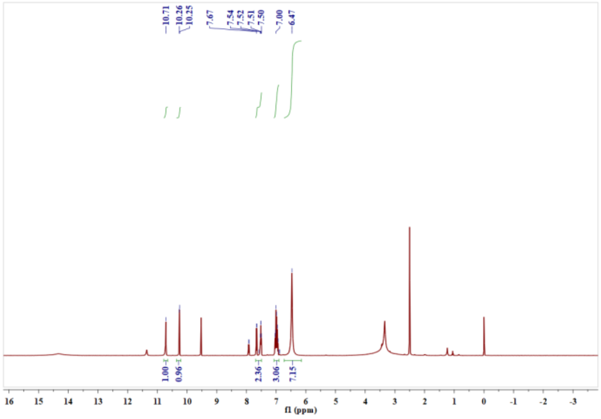

Figure S9 ^{1}H NMR (400MHz, DMSO-d_6) for **L^2H_2**.

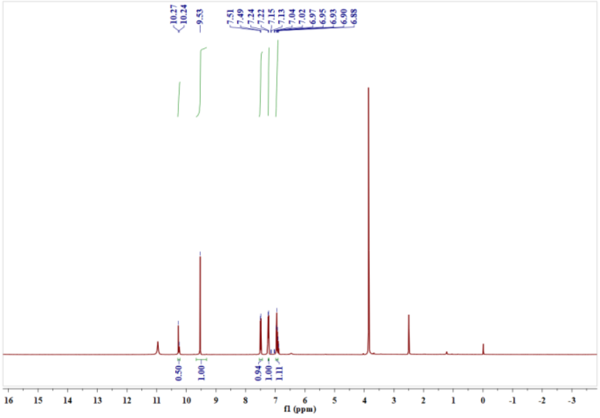

Figure S10 ^{1}H NMR (400MHz, DMSO-d$_6$) for $\mathbf{L^3H_2}$.

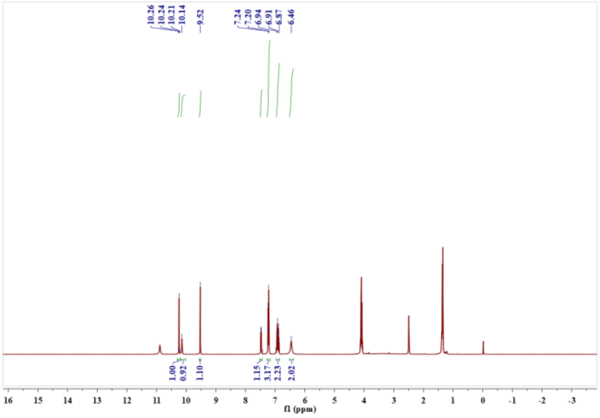

Figure S11 ^{1}H NMR (400MHz, DMSO-d$_6$) for $\mathbf{L^4H_2}$.

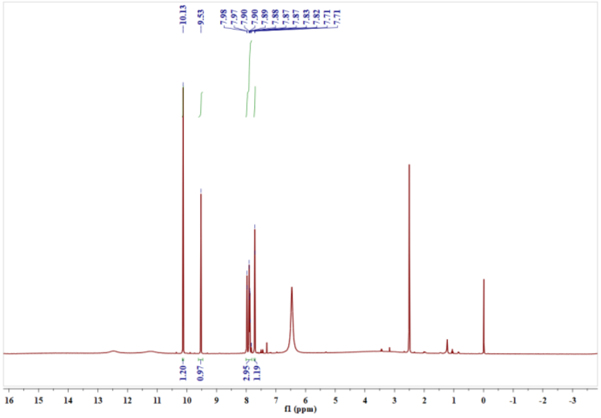

Figure S12 ^{1}H NMR (400MHz, DMSO-d$_6$) for $\mathbf{L^5H_2}$.

Supplementary Data

Electronic Supplementary Information (ESI) available: The CCDC numbers for $\mathbf{Zn_{24}}$ and 1D chain **1-D⊂**$\mathbf{Zn_{24}}$ supraclusters were 2284553 and 2284554, respectively. The data can be obtained free of charge at http://www.ccdc.cam.ac.uk, or from the Cambridge Crystallographic Data Centre, 12 Union Road, Cambridge CB21EZ, UK (Fax: +44-1223-336-033; E-mail: deposit@ccdc.cam.ac.uk).
